# The Evolution History of Fe–S Cluster A-Type Assembly Protein Reveals Multiple Gene Duplication Events and Essential Protein Motifs

**DOI:** 10.1093/gbe/evaa038

**Published:** 2020-02-27

**Authors:** Hui-Meng Lu, Jing-Di Li, Yu-Dan Zhang, Xiao-Li Lu, Chang Xu, Yuan Huang, Michael Gribskov

**Affiliations:** e1 School of Life Sciences, Key Laboratory for Space Bioscience and Biotechnology, Northwestern Polytechnical University, Xi’an, Shaanxi, PR China; e2 College of Life Sciences, Shaanxi Normal University, Xi’an, Shaanxi, PR China; e3 Department of Biological Sciences, Purdue University; e4 Department of Computer Science, Purdue University

**Keywords:** Fe–S cluster A-type assembly protein, protein family evolution, protein motif, gene duplication

## Abstract

Iron–sulfur (Fe–S) clusters play important roles in electron transfer, metabolic and biosynthetic reactions, and the regulation of gene expression. Understanding the biogenesis of Fe–S clusters is therefore relevant to many fields. In the complex process of Fe–S protein formation, the A-type assembly protein (ATAP) family, which consists of several subfamilies, plays an essential role in Fe–S cluster formation and transfer and is highly conserved across the tree of life. However, the taxonomic distribution, motif compositions, and the evolutionary history of the ATAP subfamilies are not well understood. To address these problems, our study investigated the taxonomic distribution of 321 species from a broad cross-section of taxa. Then, we identified common and specific motifs in multiple ATAP subfamilies to explain the functional conservation and nonredundancy of the ATAPs, and a novel, essential motif was found in Eumetazoa IscA1, which has a newly found magnetic function. Finally, we used phylogenetic analytical methods to reconstruct the evolution history of this family. Our results show that two types of ErpA proteins (nonproteobacteria-type ErpA1 and proteobacteria-type ErpA2) exist in bacteria. The ATAP family, consisting of seven subfamilies, can be further classified into two types of ATAPs. Type-I ATAPs include IscA, SufA, HesB, ErpA1, and IscA1, with an ErpA1-like gene as their last common ancestor, whereas type-II ATAPs consist of ErpA2 and IscA2, duplicated from an ErpA2-like gene. During the mitochondrial endosymbiosis, IscA became IscA1 in eukaryotes and ErpA2 became IscA2 in eukaryotes, respectively.

## Introduction

Iron–sulfur (Fe–S) clusters are ancient and versatile cofactors composed of iron and inorganic sulfur; they play important roles in electron transfer, metabolic and biosynthetic reactions, and the regulation of gene expression (Beinert 2000; Pain and Dancis 2016; Vernis et al. 2017). The assembly of Fe–S clusters is a highly complex and coordinated process that requires multiple cellular complexes. For example, three types of FeS assembly machinery, ISC, SUF, and NIF, have been identified in bacteria (Zheng and Santos 2018). Previous studies in bacteria have shown that NIF is involved in the maturation of nitrogenase in *Azotobacter vinelandii* (Kennedy and Dean 1992; Zheng and Dean 1994), ISC plays a housekeeping function in Fe–S cluster assembly, and SUF plays an important role under oxidative stress and iron starvation (Johnson et al. 2005; Ayala-Castro et al. 2008; Bandyopadhyay et al. 2008; Fontecave and Ollagnier-de-Choudens 2008). The ISC system is thought to have been transferred from bacteria to eukaryotes by endosymbiosis; as a result, eukaryotic mitochondria contain components homologous to the bacterial ISC system (Balk and Lobréaux 2005; Lill and Mühlenhoff 2006, 2008; Xu and Møller 2008).

A-type assembly protein (ATAP) is a conserved and essential member of the ISC, SUF, and NIF systems and plays an indispensable role in the Fe–S cluster assembly and the transfer process. The ATAP family consists of several subfamilies: IscA, in the prokaryotic ISC system; ISCA1 and ISCA2, in the eukaryotic ISC system (Lill and Kispal 2000); SufA, in the SUF system; HesB (IscA^nif^), in the NIF system (Barras et al. 2005; Fontecave et al. 2005; Johnson et al. 2005; Rubio and Ludden 2005); ErpA, which may interact with ISC and SUF (Urbina et al. 2001; Panchy et al. 2016), is essential for bacterial growth under aerobic respiratory growth conditions; and cpIscA, which is a SufA-like ATAP and was transferred from cyanobacteria to plants through endosymbiosis event, is now only harbored by plant plastids (Lill and Kispal 2000; Lu 2018). ATAPs are characterized by a conserved motif, Cys-X_n_-Cys-X-Cys (Morimoto et al. 2006), by which ATAPs can bind transiently to Fe–S clusters, presumably using the three Cys residues in this motif, and transfer the FeS cluster to apoproteins (Agar et al. 2000; Yuvaniyama et al. 2000; Urbina et al. 2001; Fontecave and Ollagnier-de-Choudens 2008).

The function of different ATAP subfamilies has been an intriguing issue for a long time. Studies have shown both redundant and nonredundant functions of different ATAPs. Most organisms contain ATAP genes from multiple ATAP subfamilies, and these genes share a similar functional domain and normally have high sequence identity. There is evidence that they can function redundantly. For example, in *Escherichia* *coli*, an *iscA sufA* double mutant was found to be conditionally lethal under aerobic growth, whereas single mutants grew almost like the wild-type strains (Lill and Kispal 2000; Lu et al. 2008; Roche et al. 2013). Findings in *Saccharomyces* *cerevisiae* also showed that a double ISA1–ISA2 knockout exhibited a mitochondrial phenotype not shown in single mutants (Kaut et al. 2000; Pelzer et al. 2000; Mühlenhoff et al. 2007). The perception that ATAPs are functionally redundant is consistent with in vitro studies in which any of the ATAPs tested were found to transfer Fe/S to apotargets with similar efficiency (Fontecave and Ollagnier-de-Choudens 2008; Panchy et al. 2016).

However, some studies also showed that ATAPs had nonredundant functions in vivo, which were likely regulated by different environmental constraints (Ollagnier-de-Choudens et al. 2004; Mo et al. 2011; Mettert and Kiley 2015). Many bacteria can harbor several ATAPs, such as HesB, SufA, IscA, and ErpA, and previous studies show that HesB can function specifically in nitrogenase formation in bacteria. SufA mainly functions under oxidative stress or iron starvation, whereas ErpA is essential under aerobic respiratory growth conditions (Loiseau et al. 2007). The nonredundant functions of ATAPs from different subfamilies can help the host better adapt to the changing environment.

**Fig. 1.**
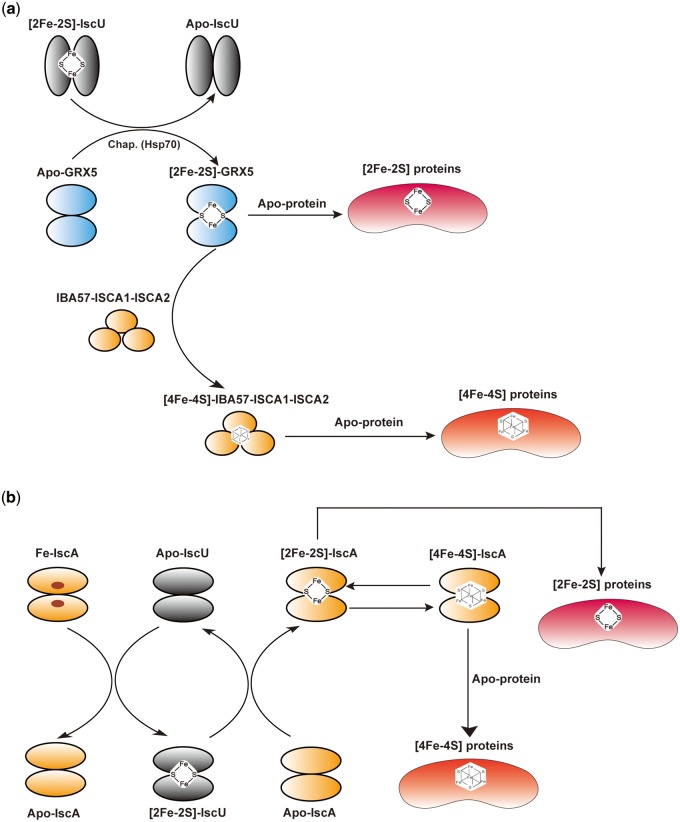
—(*a*) The main steps in the Fe–S cluster biogenesis process in eukaryotes mitochondria. Briefly, [2Fe–2S] proteins biosynthesis includes three steps: first, the [2Fe–2S] cluster assembles on the U-type protein; second, the assembled [2Fe–2S] cluster on IscU is transferred to the monothiol glutaredoxin with Hsp70 as chaperon and finally transfers to apoproteins. Further steps are needed for [4Fe–4S] proteins: two [2Fe–2S] clusters transferred by Grx5 form [4Fe–4S] cluster on the IscA1, IscA2, and Iba57 complex, which transfers the [4Fe–4S] cluster to apoproteins. (*b*) The biosynthesis of Fe–S proteins in prokaryotes by ISC pathway. First, [2Fe–2S] cluster assembles on IscU dimer with IscA as the Fe donor, then the assembled [2Fe–2S] cluster is transferred to IscA dimer and can be transferred to apoprotein to form [2Fe–2S] proteins; or [4Fe–4S] cluster can be assembled on the IscA and then be transferred to be [4Fe–4S] proteins.

Different ATAPs may interact with each other and function together during the Fe–S assembly process. For example, the two types of ATAPs, ISCA1 and ISCA2, which are found in the mitochondria of most eukaryotes, have been shown to form a complex with Iba57 or monothiol glutaredoxin 5 (GRX5), which is essential in the transfer of the [4Fe–4S] clusters or [2Fe–2S] clusters to apoproteins (Sheftel et al. 2012, p. 57; Banci et al. 2014) (fig. 1).

Currently, more findings show that apart from the Fe–S cluster transfer and assembly, ATAPs may have other functions. For example, they are possibly involved in the mechanism of Fe and Fe–S cluster sensing in some organisms (Mapolelo et al. 2013). After a novel function of IscA1 as a magnetoreceptor in *Drosophila melanogaster* was identified (Qin et al. 2016), the functional diversity of ATAP family members became even more complex. More intriguing, a previous experiment showed that yeast IscA1, but not IscA2, can be functionally replaced by an *E.* *coli* IscA, SufA, or ErpA (Mühlenhoff et al. 2011), whereas a phylogenetic study showed that eukaryotic IscA2 and proteobacteria ErpA clustered together and may share similar functions (Vinella et al. 2009).

Elucidating the evolutionary history and motif structure of the large ATAP family will help clarify the functional redundancy and specificity of each ATAP subfamily. It has been hypothesized that the ancestral ATAP gene emerged in the last common ancestor (LCA) of bacteria, after which several gene duplication events and gene transfer events have helped shape the evolutionary history of this protein family. However, to date, no study has examined the diversification of ATAP family proteins across a broad taxonomic range to illuminate the evolution and adaptation of the entire family. Both their precise function regulations and evolutionary history are still poorly understood.

Here, we investigate the distribution pattern of the ATAP family by identifying ATAP genes in 321 species/genera from all 3 domains of life. We define the motif compositions of the seven ATAP subfamilies, and common and subfamily-specific motifs are identified. Then, we associate the sequence motifs with their conserved and nonredundant functions using a functional site prediction method and structural analysis. We find two essential specific motifs in IscA1 and IscA2. The Eumetazoa IscA1-specific new motif is found to function as a linker in the formation of a magnetic complex, and the IscA2-specific functional motif may help explain why it is functionally unreplaceable by any other ATAP. Finally, our phylogenetic analysis of the ATAP family helps reconstruct the process of adaptation and evolution history of these proteins. Our results show that there are two types of ErpA proteins existing in bacteria: nonproteobacteria-type ErpA1 and proteobacteria-type ErpA2. Moreover, we demonstrate that there are two types of ATAPs. The type-I ATAP family consists of IscA, IscA1, HesB, SufA, cpIsca, and ErpA1, which may be connected with Fe–S cluster assembly scaffolds. The type-II ATAP family consists of ErpA2 and IscA2, which are predicted to interact with apoprotein targets. We suggest that IscA2 likely originated from ErpA2 and was transferred together with IscA from proteobacteria through endosymbiosis. Understanding these important evolutionary events is necessary for researchers to illustrate the important biological functions of ATAPs.

## Materials and Methods

### Data Download and Hidden Markov Model Search

To identify and classify the ATAPs across all phyla, we retrieved all the ATAP seed sequences of the Fe–S cluster assembly protein family in pfam (ID: PF01521) and generated a multiple sequence alignment (MSA) using MUSCLE v3.8.31 software (Edgar 2004). Before building the Hidden Markov Model (HMM), we manually removed non-ATAP proteins (e.g., NfuA proteins), and then the HMM profile for the ATAP family was created using hmmbuild in the HMMER3 software suite (Mistry et al. 2013, p. 3). The ATAP HMM was searched against all 9,608 reference proteomes in the UniProt database using hmmsearch in the same software suite, with an *E*-value threshold of 0.00001. The protein names in the UniProt TrEMBL database were not manually reviewed (see http://www.uniprot.org/help/protein_names, last accessed December 25, 2019); for example, we found that many sequences retrieved were labeled as HemY but turned out to be ErpA. To avoid misannotation, we then manually checked the domains of all the sequences retrieved to identify all possible ATAPs.

### Detection and Distribution of ATAPs of 321 Taxa

To better present our results, 321 completely sequenced representative species or genera from UniProt reference species were chosen from a wide range of domains based on the genomic information of the NCBI database (http://www.ncbi.nlm.nih.gov/genome/browse/, last accessed December 1, 2019), NCBI Taxonomy, and UniProt reference proteomes. Then, the ATAPs from these 321 taxa were extracted from the former hmmsearch results and annotated to their respective ATAP subfamilies. The pipeline for annotation was as follows: first, the putative ATAPs were annotated using reciprocal Blastp (Altschul et al. 1990) against the NCBI’s nonredundant protein database (Pruitt et al. 2005) and UniProtKB protein database (UniProt Consortium 2019). In this process, self-hits were excluded, and proteins annotated to be non-ATAPs were eliminated. ATAPs were annotated to specific ATAP subfamilies based on their similarity to the annotated sequences in the NCBI nonredundant database and UniProt database. However, the Blastp results with several hits were inconclusive, and some ATAPs were uncharacterized. Second, to assign subfamily-level classifications to these uncharacterized ATAPs, we generated a MSA using MUSCLE v3.8.31 software and constructed a phylogenetic tree of all the ATAPs using FastTree software (Price et al. 2009). During this process, the uncharacterized sequences were identified by their alignment similarity and clustering results with well-annotated ATAP subfamily members. Finally, we explored the EggNog 4.5.1 database (Huerta-Cepas et al. 2016) and verified the ATAP distribution pattern of some of the taxa we studied by 100% match. By utilizing this approach, we step-by-step verified and classified the ATAPs in the 321 taxa we chose.

### Motif Searches and Structural Analysis

Motifs were identified in all the ATAP subfamilies using the motif generator algorithm MEME (Bailey and Elkan 1994; Bailey et al. 2009). Several motifs identified in different subfamilies were similar to one another. Then, we mapped all the motifs generated above to all ATAPs using the MAST algorithm (Bailey and Gribskov 1998). The redundant motifs were removed from the query, and a motif was considered present in a given protein if the MAST *P* value was <10^−5^. Then, we scanned the nonredundant ATAP motifs against the protein signature database PROSITE (Sigrist et al. 2012). To further explore the function of IscA1-specific motif 6 (ATVRAVSKRKIQATR), we contacted the author (Qin et al. 2016) and obtained the original 3D structure file of the IscA1 magnetoreceptor polymer. Then, we visualized the location of the IscA1-specific motif 6 in the linear polymeric complex using PyMOL v2.3.0 (DeLano Scientific LLC., https://github.com/schrodinger/pymol-open-source, last accessed December 13, 2019). Then, we compared the structures of the bacterial IscA (*E.* *coli* IscA) and Eumetazoa IscA1 (*D.* *melanogaster* IscA1) to identify the role of motif 6 in the IscA1 tetramer. To further confirm the linking role of this motif in the formation of the magnetic linear polymeric complex in Eumetazoa, we used VMD v1.93 (Humphrey et al. 1996) and NAMD v2.11 (Phillips et al. 2005) to perform all-atom steered molecular dynamics (sMD) simulations using constant velocity stretching (SMD-CV protocol) on the combined structure of two IscA1 monomers from two neighboring IscA1 tetramers of the magnetoreceptor polymer: one monomer was fixed (except for the section of its motif 6) and the other one pulled away with the pulling speed set as 0.25 Å ns^−1^, and the maximum pulling force was calculated. Then, the similar sMD simulation on the two joining bacterial IscA monomers (PDB: 1R95) was performed.

### Multiple Alignments and Phylogenetic Analysis

To pinpoint the evolutionary history of ATAPs, we obtained representative sequences from 863 ATAPs by filtering out sequences with more than 90% identity using CD-HIT version 4.6 (Fu et al. 2012). The representative ATAPs were then aligned using MUSCLE v3.8.31 with the 5 NfuA sequence as an outgroup. Then, we used trimAl 1.4.1 (Capella-Gutiérrez et al. 2009) with the noallgaps parameter to trim the MSA automatically and removed ambiguously aligned sites manually with Jalview 2.10.4b1 (Waterhouse et al. 2009). The best model for the phylogenetic analysis was calculated by ModelFinder (Kalyaanamoorthy et al. 2017) and chosen according to the Bayesian information criterion. The maximum likelihood tree was reconstructed using IQ-TREE (Nguyen et al. 2015) using the best model selected and with the SH-aLRT test and ultrafast bootstrap (Hoang et al. 2018) with 1,000 replicates. The generated tree was depicted and submitted to iTOL (Letunic and Bork 2007) for visualization and annotation.

### The Analysis of ErpA Subfamily

To further study the duplication and diversification of bacteria and archaea *erpa* genes, we retrieved all 230 annotated ErpA protein sequences from our ATAP data set. A MSA was generated using MUSCLE v3.8.31. Then, we used trimAl 1.4.1 with the noallgaps parameter was used to trim the MSA automatically and removed ambiguously aligned sites manually using Jalview2.10.4b1. The best model for the phylogenetic analysis was calculated by ModelFinder and chosen according to the Bayesian information criterion. The maximum likelihood tree was reconstructed using IQ-TREE using the best model selected with the SH-aLRT test and ultrafast bootstrap with 1,000 replicates. The generated tree was depicted and submitted to iTOL for visualization and annotation.

## Results and Discussion

### Distribution of the A-Type Scaffold across the Tree of Life

ATAP family members were identified by searching against all 9,608 reference proteomes in the UniProt database (release 2018_01) using the HMMER profile we built. As a result, we retrieved 11,738 proteins from the 9,608 proteomes (see supplementary table S1, Supplementary Material online). After manual review and annotation, we identified 863 ATAPs from 321 chosen reference species/genera whose genomes have been completely sequenced, including 5 archaea, 178 bacteria, and 138 eukaryotes (see supplementary fig. S1, Supplementary Material online, for the taxonomy). Among the 863 ATAPs, 48 were annotated to the HesB subfamily, 136 to IscA, 182 to ISCA1, 162 to ISCA2, 59 to SufA, 230 to ErpA, and 46 to cpIscA. The retrieval and annotation results in some of our taxa have been verified by the EggNog database with a 100% match, which strongly supported the accuracy of our results in other taxa (see supplementary table S2, Supplementary Material online, for the results retrieved from the EggNog database).

Our analysis identified the presence and absence of ATAPs in all surveyed 321 taxa (see supplementary fig. S2, Supplementary Material online); overall, ATAPs are distributed widely across all the taxa, whereas certain subfamilies exist exclusively in some taxa. IscA, which has been shown to transfer from bacteria to eukaryotes through mitochondrial endosymbiosis (Lill and Mühlenhoff 2006, 2008), is universally present in all of the surveyed prokaryotes. It is located in the *isc* operon, mainly functioning in Fe–S cluster formation under general exponential aerobic growth (Ollagnier-de-Choudens et al. 2001), and its ortholog in eukaryotes, IscA1, is widely found in eukaryotes, similar to the eukaryotic IscA2. HesB, which is located in the *nif* operon and is essential for nitrogenase formation, mostly exists in nitrogen-fixing bacteria such as the genus *Rhizobium* and genus cyanobacterium. ErpA is pervasive in both bacteria and archaea but rarely found in eukaryotes, whereas SufA, which is the ATAP in the *suf* operon (Ollagnier-de Choudens et al. 2003), can be detected in archaea, bacteria, some unicellular eukaryotes such as *Plasmodium* and plant plastids (referred to as cpIscA in *Plasmodium* and plants).

Our results show that ATAPs are preferentially duplicated, which is consistent with their conserved characteristics (Davis and Petrov 2004). Multiple copies of IscA1 and IscA2 are found in some organisms, such as *Gallus gallus* and multiple flowering plants. *Gallus* *gallus* contains two copies of IscA1 (UniProt Entry: Q5ZJ74 and A0A1L1RN15), one of which lost the ATAP-specific conserved functional motif Cys-X_n_-Cys-X-Cys (UniProt Entry: A0A1L1RN15) (supplementary fig. S3, Supplementary Material online). Two to five duplicates in certain ATAP subfamilies are found in most plants we studied. For example, *Arabidopsis thaliana* and *Brassica napus*, which both belong to a large eudicot family, Brassicaceae, were found to harbor multiple ATAPs; but unlike ATAPs in *G. gallus*, these copies did not lose any functional motifs. The same results were found in multiple IscA2 copies in *Medicago truncatula* (supplementary fig. S3, Supplementary Material online). Our finding of pervasive duplicates in flowering plants is consistent with the discovery that the average flowering plant genome had nearly four rounds of ancestral genome duplication dating as far back as the common ancestor more than 300 Ma (Leebens-Mack et al. 2019), which indicates the ancient origin of ATAPs in eukaryotes.

Multiple copies of ATAPs were also found in bacteria and archaea. For example, multiple copies of IscA were found in the terrestrial clade Terrabacteria, which includes *Cyanobacteria*, two gram-positive phyla, Actinobacteria and *Firmicutes*, and two phyla with cell walls that differ structurally from typical gram-positive and gram-negative phyla, *Chloroflexi* and *Deinococcus–Thermus*. As members of this clade often possess important adaptations, such as resistance to environmental hazards (e.g., desiccation, ultraviolet radiation, and high salinity) and oxygenic photosynthesis (Lynch and Conery 2000; Seoighe and Gehring 2004; Panchy et al. 2016), we hypothesize that these features of Terrabacteria might help explain the retention of the IscA duplicates as this protein can be beneficial for bacterial adaptation. Meanwhile, most members of Betaproteobacteria that we studied also possess more than one IscA, and their ErpA is also duplicated pervasively (see supplementary fig. S2, Supplementary Material online). The identical copy numbers of IscA and ErpA indicate that these species in Betaproteobacteria might have experienced large genome fragment duplication when these two genes (*iscA* and *erpa*) duplicated together.

### Gain and Loss of Motifs during the Evolution of ATAPs

Motifs are highly similar regions among protein sequences and may represent sites of protein–protein interactions and posttranslational modification. A previous study showed that members from different ATAP subfamilies performed both redundant and nonredundant functions under different conditions, even though their sequences shared high similarity (Ollagnier-de-Choudens et al. 2001; Ollagnier-de Choudens et al. 2003; Loiseau et al. 2007). The C-terminal tail of ATAPs, which consist of two Cys residues, is a highly conserved motif in the ATAP family. However, the N-terminal and middle segments of the ATAP sequences are variable in length and intervals. The analysis of differential gain and loss of motifs can improve our understanding of ATAP subfunctionalization and conservation.

**Fig. 2.**
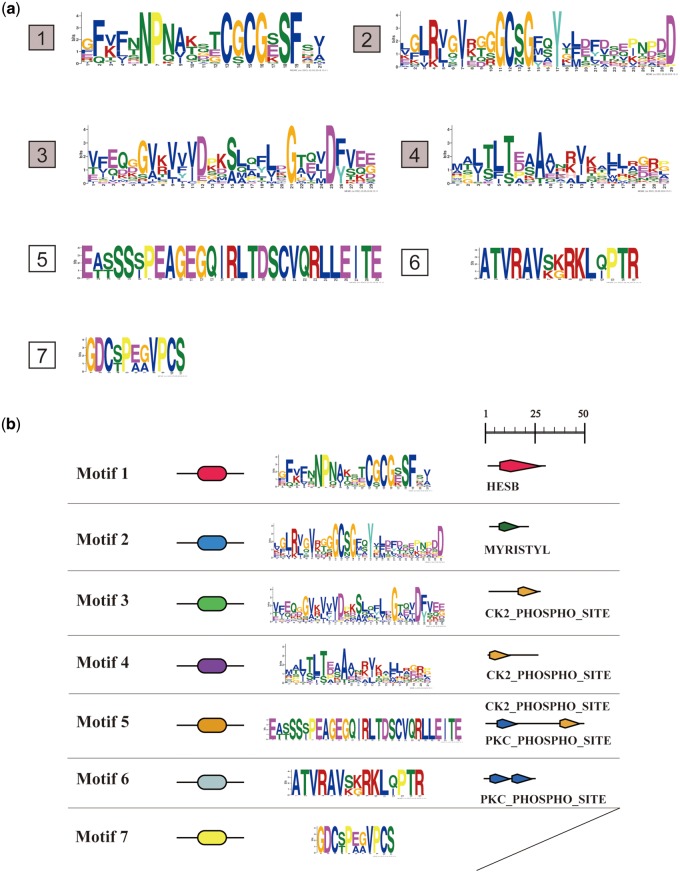
—(*a*) The seven unique motifs generated by MEME algorithm, the first four motifs were core motifs 1–4, which were present in almost all ATAPs. Motifs 5–7 were lineage-specific motifs. Motif 5 is an IscA2-specific motif, which is present widely in eukaryotes. Motif 6 is an IscA1-specific N-terminal motif and is present widely in the Eumetazoa. Motif 7 is a HesB C-terminal hallmark motif. (*b*) The length and signature characteristics of each motif, the right side of each motif presented the functional site found using PROSITE. Motif 1, hypothetical hesB/yadR/yfhF family signature; motif 2, N-myristoylation site; motif 3, casein kinase II phosphorylation site; motif 4, casein kinase II phosphorylation site; motif 5, protein kinase C phosphorylation site, casein kinase II phosphorylation site; motif 6, protein kinase C phosphorylation site.

**Fig. 3.**
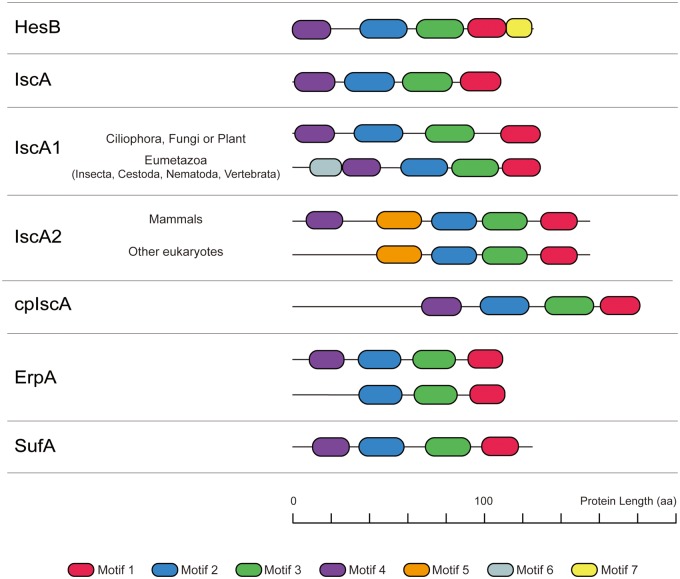
—Motif characterization and sequence length among the seven ATAP subfamilies. Motifs 1–4 are present in all the ATAPs with similar arrangements, motif 6 is widely present in Eumetazoa IscA1 and located in the N-terminal of the sequence, motif 5 is an IscA2-specific motif, and motif 7 is a HesB-specific motif.

In our study, seven unique motifs were identified in the ATAP family (fig. 2). Multiple motifs (motifs 1–4) were found common across different ATAP subfamilies, which is consistent with their functional conservation (fig. 3). Specific motifs were also detected in different subfamilies. For example, IscA2-specific motif 5, IscA1-specific motif 6, and HesB-specific motif 7 were detected. Although non-Eumetazoa IscA1 and ErpA contain four identical motifs (motifs 1–4), these motifs showed different patterns. We also detected the motif loss in some ErpA duplicates in bacteria; many bacteria contain two copies of ErpA, and sometimes the N-terminal motif 4 of one copy of ErpA was lost (fig. 3).

The following conclusions can be drawn from our motif analysis. First, motifs 1–4 are highly conserved in every ATAP and are rarely lost, which suggests they perform a function conserved among all ATAPs. Motif 1 is located in the C-terminal tail of ATAPs and contains two Cys residues that are essential in Fe–S cluster binding; in addition, motif 1 is the signature motif of the hesB/yadR/yfhF family (Morimoto et al. 2006). Motif 2 contains an N-terminal Cys residue that is thought to play a core function in binding Fe–S clusters, along with the other two Cys residues contained in motif 1 (Agar et al. 2000; Yuvaniyama et al. 2000; Urbina et al. 2001; Fontecave and Ollagnier-de-Choudens 2008). Motifs 3 and 4 contain casein kinase II phosphorylation sites, which indicates that the posttranslational modification and signal transduction of ATAPs may be related to the largely uncharacterized functions of motifs 3 and 4. We further found that several ATAP paralogs have gained “new” N-terminal motifs. For example, in Eumetazoa (fig. 3), we have identified motif 6 in the IscA1 N-terminal tail, which might be involved in the formation of an IscA1-involved magnetic biocompass. In eukaryotic IscA2, we found a new middle motif 5. Motif 5 is the longest among all seven motifs and contains both a predicted casein kinase II phosphorylation site and protein kinase C phosphorylation sites. This IscA2-specific motif 5 must play a specific function, which can be used to explain the irreplaceability of yeast IscA2 (Seo and Lee 2004; Mühlenhoff et al. 2011). Motif 7 is a HesB-specific motif found in the C-terminal end of the protein and is a hallmark for HesB proteins.

**Fig. 4.**
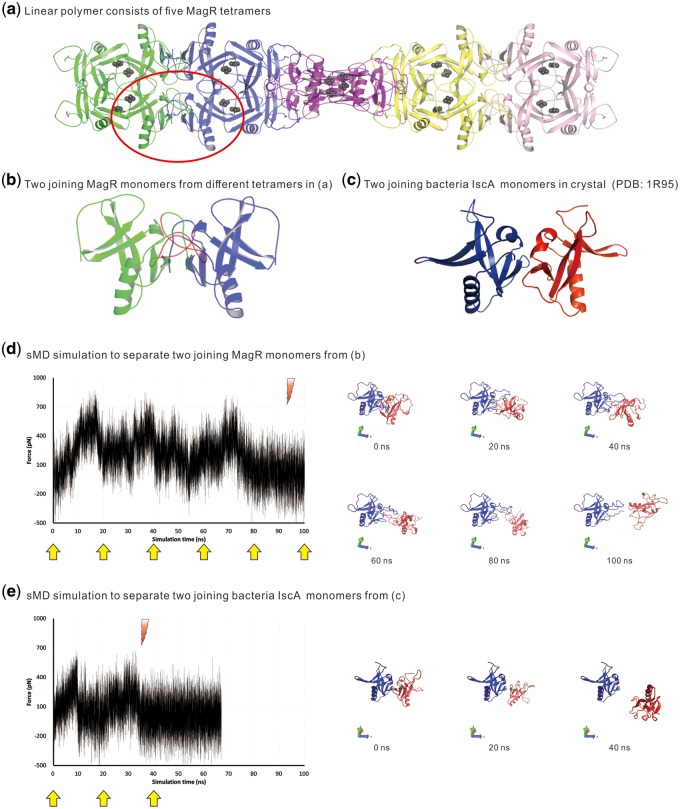
—(*a*) The structure of IscA1-composed magnetic polymer without the Cry. Two IscA1 first form dimers via intermolecular interactions, then, two IscA1 dimers form a functional tetramer with Fe located in the active center of the tetramer. Then, the tetramers form a long chain of protein complex by intermolecular interactions. The two joining IscA1 monomers between neighboring tetramers were labeled by oval-shaped red line. (*b*) The enlarged description of the two joining IscA1 monomers labeled by the red oval in (*a*). The IscA1-specific motif 6 (colored in red) were shown to be located in the junction surface between the two neighboring IscA1 tetramers. (*c*) The 3D structure of the two joining bacterial IscA monomers (PDB: 1R95) without motif 6 showed that the two monomers did not link with each other. (*d*) The result of sMD simulation using the SMD-CV protocol on the structure in (*b*). The left-side plot showed the fluctuation of the pulling force during the sMD, in which the maximum pulling force increased up to 871 pN. The 3D structures retrieved at several checkpoints (labeled by the yellow arrows) were shown on the right side which showed that the two IscA1 monomers were separated after pulling for about 90 ns (labeled by the red arrow in the left-side plot). (*e*) The result of the sMD simulation using the SMD-CV protocol on the combined structure of the two bacterial IscA monomers in (*c*). The left-side plot showed the fluctuation of the pulling force during the sMD, in which the maximum pulling force increased up to 684 pN. The 3D structures retrieved at several checkpoints (labeled by the yellow arrows) were shown on the right side which showed that the two IscA monomers were separated after pulling for about 35 ns (labeled by the red arrow in the left-side plot).

The N-terminus of IscA1 containing motif 6 had been shown to interact with Cryptochrome (Cry) to form a light-magnetic sensor, and the formation of IscA1 linear polymer was necessary for the magnetic compass function (Qin et al. 2016; Zhou et al. 2016). Our structural visualization results showed that motif 6 was located in the junction surface between neighboring IscA1 tetramers within the linear polymer of magnetoreceptor (*D.* *melanogaster* IscA1). In the meantime, the bacterial IscA, which did not contain motif 6, had never been shown to form a linear polymer (fig. 4*a*–*c*). This result suggested that the IscA1-specific motif 6 might play an essential role in the formation of the magnetic compass in Eumetazoa.

By further conducting the sMD simulation on the combined structure of two IscA1 monomers and two IscA monomers (fig. 4*d* and *e*), we found that with the motif 6, the two joining IscA1 monomers can form a tighter junction than that in the two IscA monomers (fig. 4*d* and *e*), which led to the successful formation of the magnetic polymer. It took us about 90 ns to separate the two linking IscA1 monomers when we tried to pull one IscA1 monomer from the other and the maximum pulling force increased up to 871 pN (fig. 4*d*). However, it only took us about 35 ns to separate the two joining bacterial IscA monomers and the maximum force of sMD increased to 684 pN, which was smaller than that in the two IscA1 monomers, too (fig. 4*e*). The sMD simulation results further confirmed that the IscA1 N-terminal motif 6 played an important role as a hook to concatenate two neighboring IscA1 tetramers in the magnetoreceptor polymer.

### The Evolutionary History of ATAPs

**Fig. 5.**
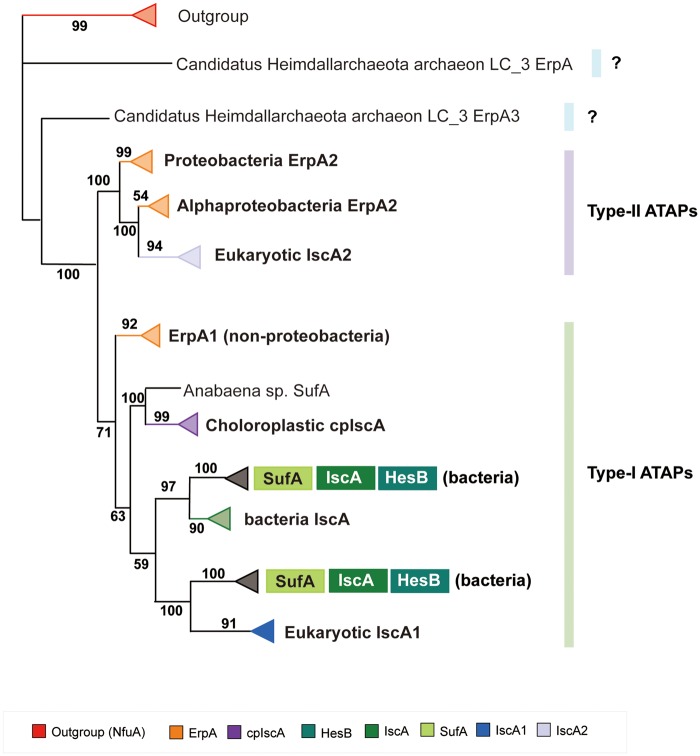
—The annotated collapsed phylogeny of the ATAP family and an outgroup (NfuA). Different ATAP subfamilies have been indicated by the triangles filled with different color and the bootstrap value for each clade was labeled. There were mainly two clades found, indicating the type-I ATAP family and the type-II ATAP family, respectively. In the type-I ATAP family, nonproteobacteria ErpA1 clustered with the other five ATAP subfamilies (eukaryotic IscA1, HesB, SufA, prokaryotic IscA, and cpIscA). In the type-II ATAP family, proteobacteria ErpA2 formed a separate clade with eukaryotic IscA2. There was also a branch of archaea-type ErpA3 located distantly from the two major types of ATAPs.

A total of 555 representative sequences were retrieved from 863 ATAPs. Then, the MSA was generated and refined for these 555 sequences and the outgroup (5 NfuA sequences) (see supplementary table S4, Supplementary Material online). According to the BIC, the best model for constructing their phylogeny was VT + R10 (see supplementary table S5, Supplementary Material online). Then, the maximum likelihood tree was reconstructed with the VT + R10 model, and the phylogeny of the ATAPs showed a number of monophyletic groups, most of which were well supported (bootstrap values can be referred on the branch), with each clade corresponding to an ATAP subfamily (see supplementary fig. S4, Supplementary Material online). Our phylogenetic analysis shows that IscA, IscA1, HesB, SufA, cpIsca, and ErpA of nonproteobacteria group phyla (including FCB group, Terrabacteria group, and PVC group) are clustering together, whereas eukaryotic IscA2 forms a separate clade with the proteobacteria ErpA (fig. 5). Moreover, some archaea ATAPs were located distantly with bacteria and eukaryotes. For example, two ErpA copies of Candidatus Heimdallarchaeota archaeon LC_3 did not cluster with any other ATAPs. To further explore the evolution of the ErpA subfamily, we conducted phylogenetic analysis of the 230 ErpA sequences. The best model calculated for the MSA (see supplementary table S6, Supplementary Material online) of ErpAs was LG + R10 (see supplementary table S7, Supplementary Material online), and the result phylogeny showed that bacteria ErpAs mainly separate into two clades that correspond to proteobacteria phyla and nonproteobacteria phyla (including the phylum Firmicutes and phylum Actinobacteria), respectively (with a bootstrap support of 84) (see supplementary fig. S5, Supplementary Material online). Multiple copies of ErpA from the same bacterium were found to cluster together in the same clade. We found that an archaea ErpA clustered with the proteobacteria clade ErpA (ErpA of Candidatus Heimdallarchaeota archaeon LC_3 clustered with ErpA of the Pelagibacterales bacterium TMED287 with a bootstrap support 99), whereas the other two ErpA copies of the same archaea were located distantly from bacterial ErpAs. These results indicate that there are more than two types of ErpA proteins, nonproteobacteria-type ErpA (referred to as ErpA1 here), proteobacteria-type ErpA (referred to as ErpA2 here), and archaea-type ErpA (referred to as ErpA3). ErpA1 exists in nonproteobacteria and ErpA2 exists in proteobacteria, whereas both ErpA2 and ErpA3 were found in archaea. Because there are few functional studies on archaea, our main focus in this study is on ErpA1 and ErpA2.

The result that ErpA2 forms an independent clade with eukaryotic IscA2 rather than with the other five ATAP subfamilies seems to contradict previous evidence that eukaryotic IscA1 can be functionally replaced by *E.* *coli* ErpA2, IscA, and SufA, whereas eukaryotic IscA2 cannot. After further investigation, we found that ErpA2 and eukaryotic IscA2 shared higher sequence similarity and IscA2 can interact with IscA1 in the eukaryotic ISC system, resembling the partnership between ErpA2/IscA or ErpA2/SufA in bacteria (Vinella et al. 2009). A study has shown that IscA and SufA can transfer Fe–S clusters to ErpA2, and then ErpA2 can deliver the Fe–S clusters to apoprotein targets. IscA and SufA are connected with Fe–S assembly scaffolds, whereas ErpA2 is predicted to interact with apotargets (Py et al. 2018). Thus, IscA2 is likely to play a similar role as ErpA2 in the maturation of Fe–S proteins (Pelzer et al. 2000; Gelling et al. 2008; Mühlenhoff et al. 2011; Brancaccio et al. 2014; Beilschmidt et al. 2017). Therefore, we classified two families of ATAPs, the type-I ATAP family consisting of IscA, IscA1, HesB, SufA, cpIsca, and ErpA1, which are connected with Fe–S cluster assembly scaffolds, and the type-II ATAP family consisting of ErpA2 and IscA2, which interact with apoprotein targets, and we suggested that the archaea-type ErpA3 did not belong to these two ATAP families. We suggested that IscA2 likely originated from ErpA2 and was transferred together with IscA1 from proteobacteria through endosymbiosis. Then, IscA2 went through some mutations and received a new important functional motif (motif 5 from our previous result), which made it unreplaceable by neither its ortholog ErpA2 nor the other ATAPs.

Our results also show that compared with members from other ATAP subfamilies, ErpAs are located closer to the ancestral node of the phylogeny. ErpA1 is located at the ancestral node of the ATAP type-I clade, and ErpA2 is located at the ancestral node of the ATAP type-II clade. The result that ErpA is distributed across both the type-I and type-II families of ATAP suggests that the LCA of ATAP is likely to be an ErpA-like gene that has undergone gene duplication and generated two types of ErpA in bacteria.

**Fig. 6.**
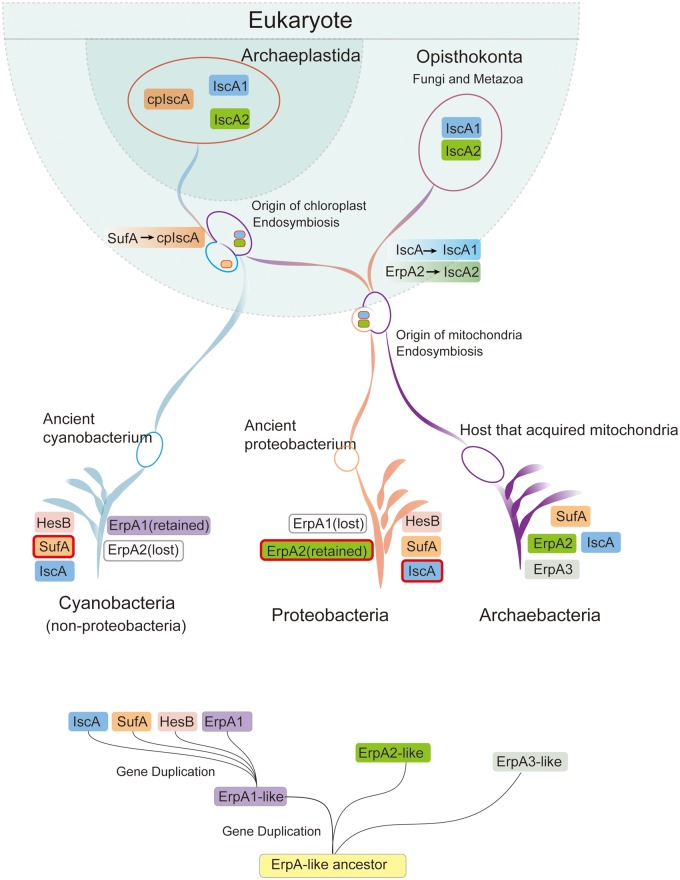
—Evolutionary history inferred for the ATAP family. The LCA of the entire ATAP family was likely an ErpA-like gene. In the first round of gene duplication event, the ancestor ErpA-like gene duplicated into an ErpA1-like gene, an ErpA2-like gene and presumably an ErpA3-like gene which can only be found in archaea now and is waiting to be explored. After the divergence of the major bacterial groups, both the proteobacteria and nonproteobacteria contain both the ErpA1-like and ErpA2-like genes. Then, the ErpA1-like gene duplicated into ErpA1, IscA, SufA, and HesB in the second round of gene duplication event. Before the endosymbiosis, several gene loss events happened in prokaryotes through which proteobacteria lost the ErpA1 and nonproteobacteria lost ErpA2, this explains why nowadays we can only detect one type of ErpA in these two major prokaryotic groups. Then, the ATAPs were transferred from proteobacteria and cyanobacteria through endosymbiosis of mitochondria and chloroplast, after which the IscA1 (from prokaryotic IscA) and IscA2 (from prokaryotic ErpA2) were harbored by eukaryotic mitochondria and cpIscA (from prokaryotic SufA) was harbored by the plant plastids. How the archaea-type ErpA3 evolved during the time is waiting to be explored.

Collectively, we suggest that the entire ATAP family has evolved through two rounds of gene duplication as well as endosymbiosis (Cózar-Castellano et al. 2004; Abdel-Ghany et al. 2005). The LCA of ATAP is likely to be an ErpA-like gene existing in ancient prokaryotes (the ancestor of bacteria and archaea). After the first round of gene duplication, two types of ErpA (nonproteobacteria-type ErpA1-like gene and proteobacteria-type ErpA2-like gene) were produced, and then IscA HesB and SufA duplicated from the ErpA1-like gene and occurred in the second round of gene duplication event (fig. 6). Whether archaea-type ErpA3 was generated during the first or second round of gene duplication remains to be explored. When the major bacteria groups diverged ∼2.5–3.2 Ga (Battistuzzi et al. 2004), both the proteobacteria group and nonproteobacteria group contained the two types of ErpA-like genes, and then through two rounds of gene duplication events and mitochondria endosymbiosis, the ErpA1-like gene duplicated to IscA and transferred, becoming eukaryotic IscA1, and the ErpA2-like gene transferred, becoming IscA2. However, during the later evolutionary period of bacteria, only ErpA1 was retained in the nonproteobacteria phyla, and ErpA2 was retained in the proteobacteria phyla.

## Conclusion

Here, we first mined the genomes of thousands of organisms spanning the tree of life to classify and identify seven ATAP subfamilies. We used HMMER software to detect the distribution of ATAP family members among all 9,608 UniProt reference species, and we chose 321 representative species/genera to better present our results. We identified the common and specific motifs of members of the each ATAP subfamily to explain their functional conservation and nonredundancy. Furthermore, we conducted structural analysis of the IscA1-specific motif and revealed that it functioned as a hook to connect the IscA1 tetramers and helped them form a magnetoreceptor complex. These results may also shed a light on how to find more useful protein motifs as mechanical joints in the design of molecular nanorobots (Huang et al. 2016). We have also constructed a comprehensive phylogeny of ATAPs and retraced the comprehensive evolutionary history of this large and complex protein family. Our study identified two types of ErpA in bacteria: nonproteobacteria-type ErpA1 and proteobacteria-type ErpA2. They likely originated from the ErpA-like ancestor ATAP gene and went through two rounds of gene duplication events to become IscA, SufA, HesB, ErpA1, and ErpA2 in the ancient prokaryotes. During the mitochondrial endosymbiosis, IscA became IscA1 in eukaryotes, and ErpA2 became IscA2 in eukaryotes. Our results suggest that these seven ATAP subfamilies can be classified into two families. The type-I ATAP family that originated from an ErpA1-like ancestor gene and includes IscA, IscA1, HesB, SufA, cpIsca, and ErpA1 are connected with Fe–S cluster assembly scaffolds, and the type-II ATAP family that originated from an ErpA2-like ancestor gene and consists of ErpA2 and IscA2 can interact with apoprotein targets. We also found that there was an archaea-type ErpA3 that did not belong to these two ATAP families.

## Supplementary Material

evaa038_Supplementary_Data

## Notes

**Data deposition:** This project has been deposited at FigShare under the accession https://doi.org/10.6084/m9.figshare.11847183 (last accessed February 13, 2020).
