# Supplementary material for: The Evolution History of Fe–S Cluster A-Type Assembly Protein Reveals Multiple Gene Duplication Events and Essential Protein Motifs
Source: Genome Biol Evol. 2020 Feb 27;12(3):160–73. doi: 10.1093/gbe/evaa038 (PMC7144353; doi:10.1093/gbe/evaa038)
Supplement: evaa038_Supplementary_Data [file evaa038_supplementary_data.zip › Supplementary_LegendAndFig.pdf]

Supplementary Materials

Supplementary Table S1: 11738 entries retrieved from all the uniport reference proteomes using hmmsearch.

Supplementary Table S2: Taxa retrieved from EggNog database with corresponding ATAP distributions (this was used for validation of part of our results in 321 representative species, which was in Fig. S3).

Supplementary Table S3: Motif patterns of ATAP sequences detected by MAST algorism.

Supplementary Table S4: The multiple sequence alignment of the ATAP family (555 representative ATAP sequences and 5 NfuA sequences as an outgroup).

Supplementary Table S5: The best model selected according to BIC for the phylogenetic reconstruction of the ATAP family.

Supplementary Table S6: The multiple sequence alignment of the ErpA family (230 ErpA sequences).

Supplementary Table S7: The best model selected according to BIC for the phylogenetic reconstruction of the ErpA family.

Supplementary Figure S1: The taxonomy of all the 321 representative species/genera we studied, including 5 archaea, 138 eukaryotes and 178 bacteria.

Supplementary Figure S2: The overall distribution of the seven ATAP subfamilies (HesB, IscA, IscA1, IscA2, cpIscA, ErpA and SufA) in the 321 representative species/genera we studied, the presence of an ATAP in a species was shown by a point. Different ATAP subfamilies were indicated by different color of the points.

Supplementary Figure S3: The sequence alignment of multiple ATAP copies harboured in *Gallus gallus*, *Arabidopsis thaliana* and *Brassica napus*, and the conserved ATAP motif Cys-Xn-Cys-X-Cys was marked by red round frame. a. The alignment of two copies of IscA1 in *G. gallus*, one of the copies lost the conserved motif; b. The alignment of the two IscA1 in *A. thaliana*, both IscA1 sequences contained the conserved motif; c. The multiple alignment of the four copies of cpIscA, two copies of IscA1 and two copies of IscA2, the conserved motif showed no sign of being lost in these duplicates.

Supplementary Figure S4: The phylogenetic tree of the entire ATAP family with bootstrap values on the branches. It was reconstructed using the representative 555 ATAP sequences, with 5 NfuA sequences as an outgroup (the outgroup clade was labeled red). Clades representing monophyletic ATAP subfamilies have been labeled with different color and text annotations.

Supplementary Figure S5: The phylogenetic tree of ErpA subfamily with bootstrap values on the branches. The major two groups of prokaryotic ErpA have been labeled with different colors and text annotations. There was also an archaea-type ErpA3 located distantly from the two major groups.

Figure S1

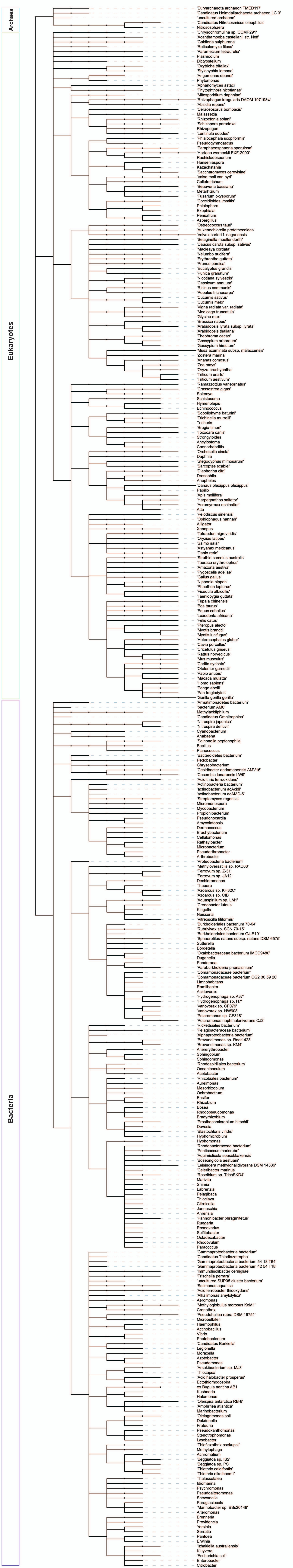
Archaea
Eukaryotes
Bacteria
'Euryarchaeota archaeon TMED117'
'Candidatus Heimdallarchaeota archaeon LC 3'
'uncultured archaeon'
'Candidatus Nitrocosmicus oleophilus'
Nitrososphaera
'Chrysochromulina sp. CCMP291'
'Acanthamoeba castellanii str. Neff'
'Galdieria sulphuraria'
'Reticulomyxa filosa'
'Paramecium tetraurelia'
Plasmodium
Dictyostelium
'Oxytricha trifallax'
'Stylonychia lemnae'
'Angomonas deanei'
Phytomonas
'Aphanomyces astaci'
'Phytophthora nicotianae'
'Mitosporidium daphniae'
'Rhizophagus irregularis DAOM 197198w'
'Absidia repens'
'Ceraceosorus bombacis'
Malassezia
'Rhizoctonia solani'
'Schizopora paradoxa'
Rhizopogon
'Lentinula edodes'
'Phialocephala scopiformis'
Pseudogymnoascus
'Paraphaeosphaeria sporulosa'
'Hortaea werneckii EXF-2000'
Rachicladosporium
Hanseniaspora
Kazachstania
'Saccharomyces cerevisiae'
'Valsa mali var. pyri'
Colletotrichum
'Beauveria bassiana'
Metarhizium
'Fusarium oxysporum'
'Coccidioides immitis'
Phialophora
Exophiala
Penicillium
Aspergillus
'Ostreococcus tauri'
'Auxenochlorella protothecoides'
'Volvox carteri f. nagariensis'
'Selaginella moellendorffii'
'Daucus carota subsp. sativus'
'Macleaya cordata'
'Nelumbo nucifera'
'Erythranthe guttata'
'Prunus persica'
'Eucalyptus grandis'
'Punica granatum'
'Nicotiana sylvestris'
'Capsicum annuum'
'Ricinus communis'
'Populus trichocarpa'
'Cucumis sativus'
'Cucumis melo'
'Vigna radiata var. radiata'
'Medicago truncatula'
'Glycine max'
'Brassica napus'
'Arabidopsis lyrata subsp. lyrata'
'Arabidopsis thaliana'
'Theobroma cacao'
'Gossypium arboreum'
'Gossypium hirsutum'
'Musa acuminata subsp. malaccensis'
'Zostera marina'
'Ananas comosus'
'Zea mays'
'Oryza brachyantha'
'Triticum urartu'
'Triticum aestivum'
'Ramazzottius varieornatus'
'Crassostrea gigas'
Solemya
Schistosoma
Hymenolepis
Echinococcus
'Soboliphyme baturini'
'Trichinella murrelli'
Trichuris
'Brugia timori'
'Toxocara canis'
Strongyloides
Ancylostoma
Caenorhabditis
'Orchesella cincta'
Daphnia
'Stegodyphus mimosarum'
'Sarcoptes scabiei'
'Diaphorina citri'
Drosophila
Anopheles
'Danaus plexippus plexippus'
Papilio
'Apis mellifera'
'Harpegnathos saltator'
'Acromyrmex echinatior'
Atta
'Pelodiscus sinensis'
'Ophiophagus hannah'
Alligator
Xenopus
'Tetraodon nigroviridis'
'Oryzias latipes'
'Salmo salar'
'Astyanax mexicanus'
'Danio rerio'
'Struthio camelus australis'
'Tauraco erythrolophus'
'Amazona aestiva'
'Pygoscelis adeliae'
'Gallus gallus'
'Nipponia nippon'
'Phaethon lepturus'
'Ficedula albicollis'
'Taeniopygia guttata'
'Tupaia chinensis'
'Bos taurus'
'Equus caballus'
'Loxodonta africana'
'Felis catus'
'Pteropus alecto'
'Myotis brandtii'
'Myotis lucifugus'
'Heterocephalus glaber'
'Cavia porcellus'
'Cricetulus griseus'
'Rattus norvegicus'
'Mus musculus'
'Carlito syrichta'
'Otolemur garnettii'
'Papio anubis'
'Macaca mulatta'
'Homo sapiens'
'Pongo abelii'
'Pan troglodytes'
'Gorilla gorilla gorilla'
'Armatimonadetes bacterium'
'bacterium AM6'
Methylacidiphilum
'Candidatus Omnitrophica'
'Nitrospira japonica'
'Nitrospira defluvii'
Cyanobacterium
Anabaena
'Seinonella peptonophila'
Bacillus
Planococcus
'Bacteroidetes bacterium'
Pedobacter
Chryseobacterium
'Cesiribacter andamanensis AMV16'
'Cecembia lonarensis LW9'
'Acidithrix ferrooxidans'
'Actinobacteria bacterium'
'actinobacterium acAcidi'
'actinobacterium acAMD-5'
'Streptomyces regensis'
Micromonospora
Mycobacterium
Propionibacterium
Pseudonocardia
Amycolatopsis
Dermacoccus
Brachybacterium
Cellulomonas
Rathayibacter
Microbacterium
Pseudarthrobacter
Arthrobacter
'Proteobacteria bacterium'
'Methyloversatilis sp. RAC08'
'Ferrovum sp. Z-31'
'Ferrovum sp. JA12'
Dechloromonas
Thauera
'Azoarcus sp. KH32C'
'Azoarcus sp. CIB'
'Aquaspirillum sp. LM1'
'Crenobacter luteus'
Kingella
Neisseria
'Vitreoscilla filiformis'
'Burkholderiales bacterium 70-64'
'Rubrivivax sp. SCN 70-15'
'Burkholderiales bacterium GJ-E10'
'Sphaerotilus natans subsp. natans DSM 6575'
Sutterella
Bordetella
'Oxalobacteraceae bacterium IMCC9480'
Duganella
Pandoraea
'Paraburkholderia phenazinium'
'Comamonadaceae bacterium'
'Comamonadaceae bacterium CG2 30 59 20'
Limnohabitans
Ramlibacter
Acidovorax
'Hydrogenophaga sp. A37'
'Hydrogenophaga sp. H7'
'Variovorax sp. CF079'
'Variovorax sp. HW608'
'Polaromonas sp. CF318'
'Polaromonas naphthalenivorans CJ2'
'Rickettsiales bacterium'
'Pelagibacteraceae bacterium'
'Alphaproteobacteria bacterium'
'Brevundimonas sp. Root1423'
'Brevundimonas sp. KM4'
Altererythrobacter
Sphingobium
Sphingomonas
'Rhodospirillales bacterium'
Oceanibaculum
Acetobacter
'Rhizobiales bacterium'
Aureimonas
Mesorhizobium
Ochrobactrum
Ensifer
Rhizobium
Bosea
Rhodopseudomonas
Bradyrhizobium
'Prosthecomicrobium hirschii'
Devosia
'Blastochloris viridis'
Hyphomicrobium
Hyphomonas
'Rhodobacteraceae bacterium'
'Ponticoccus marisrubri'
'Aquimixticola soesokkakensis'
'Boseongicola aestuarii'
'Leisingera methylohalidivorans DSM 14336'
'Celeribacter marinus'
'Roseibium sp. TrichSKD4'
Marivita
Shimia
Labrenzia
Pelagibaca
Thioclava
Citreicella
Jannaschia
Ahrensia
'Pannonibacter phragmitetus'
Ruegeria
Roseovarius
Sulfitobacter
Octadecabacter
Rhodovulum
Paracoccus
'Gammaproteobacteria bacterium'
'Candidatus Thiodiazotropha'
'Gammaproteobacteria bacterium 54 18 T64'
'Gammaproteobacteria bacterium 42 54 T18'
'Immundisolibacter cernigliae'
'Frischella perrara'
'uncultured SUP05 cluster bacterium'
'Solimonas aquatica'
'Acidiferrobacter thiooxydans'
'Alkalimonas amylolytica'
Aeromonas
'Methyloglobulus morosus KoM1'
Crenothrix
'Pseudohaliea rubra DSM 19751'
Microbulbifer
Haemophilus
Actinobacillus
Vibrio
Photobacterium
'Candidatus Berkiella'
Legionella
Moraxella
Azotobacter
Pseudomonas
'Arsukibacterium sp. MJ3'
Thiocapsa
'Acidihalobacter prosperus'
Ectothiorhodospira
ex Bugula neritina AB1
Kushneria
Halomonas
'Oleispira antarctica RB-8'
'Amphritea atlantica'
Marinobacterium
'Oleiagrimonas soli'
Dokdonella
Frateuria
Pseudoxanthomonas
Stenotrophomonas
Lysobacter
'Thioflexothrix psekupsii'
Methylophaga
Achromatium
'Beggiatoa sp. IS2'
'Beggiatoa sp. PS'
'Thiothrix caldifontis'
'Thiothrix eikelboomii'
Thalassotalea
Idiomarina
Psychromonas
Pseudoalteromonas
Shewanella
Paraglaciecola
'Marinobacter sp. BSs20148'
Alteromonas
Brenneria
Providencia
Yersinia
Serratia
Pantoea
Erwinia
'Izhakiella australiensis'
Kluyvera
'Escherichia coli'
Enterobacter
Citrobacter

Figure S2

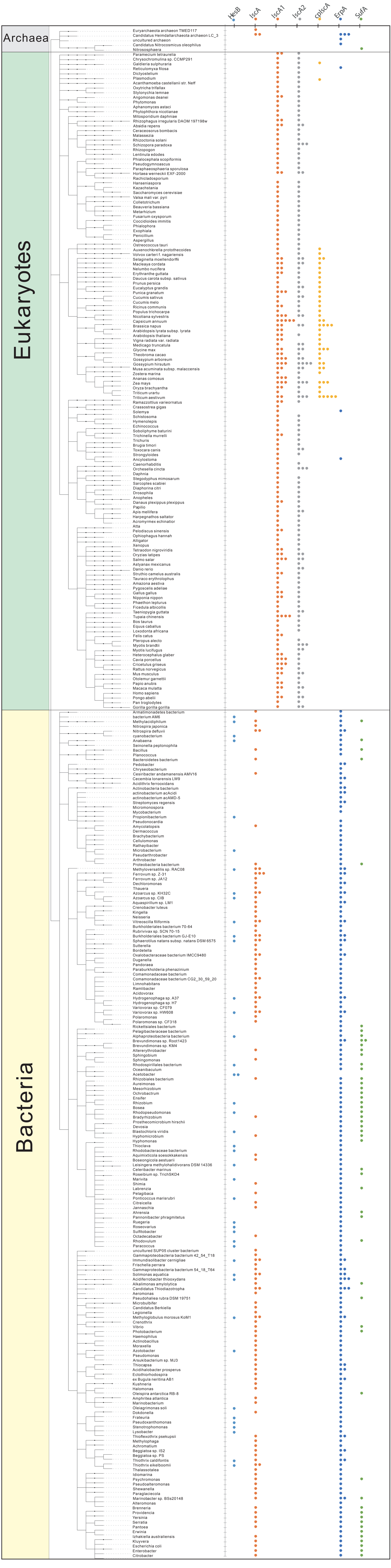
HesB
IscA
IscA1
IscA2
cpIscA
ErpA
SufA
Archaea
Eukaryotes
Bacteria

Figure S3

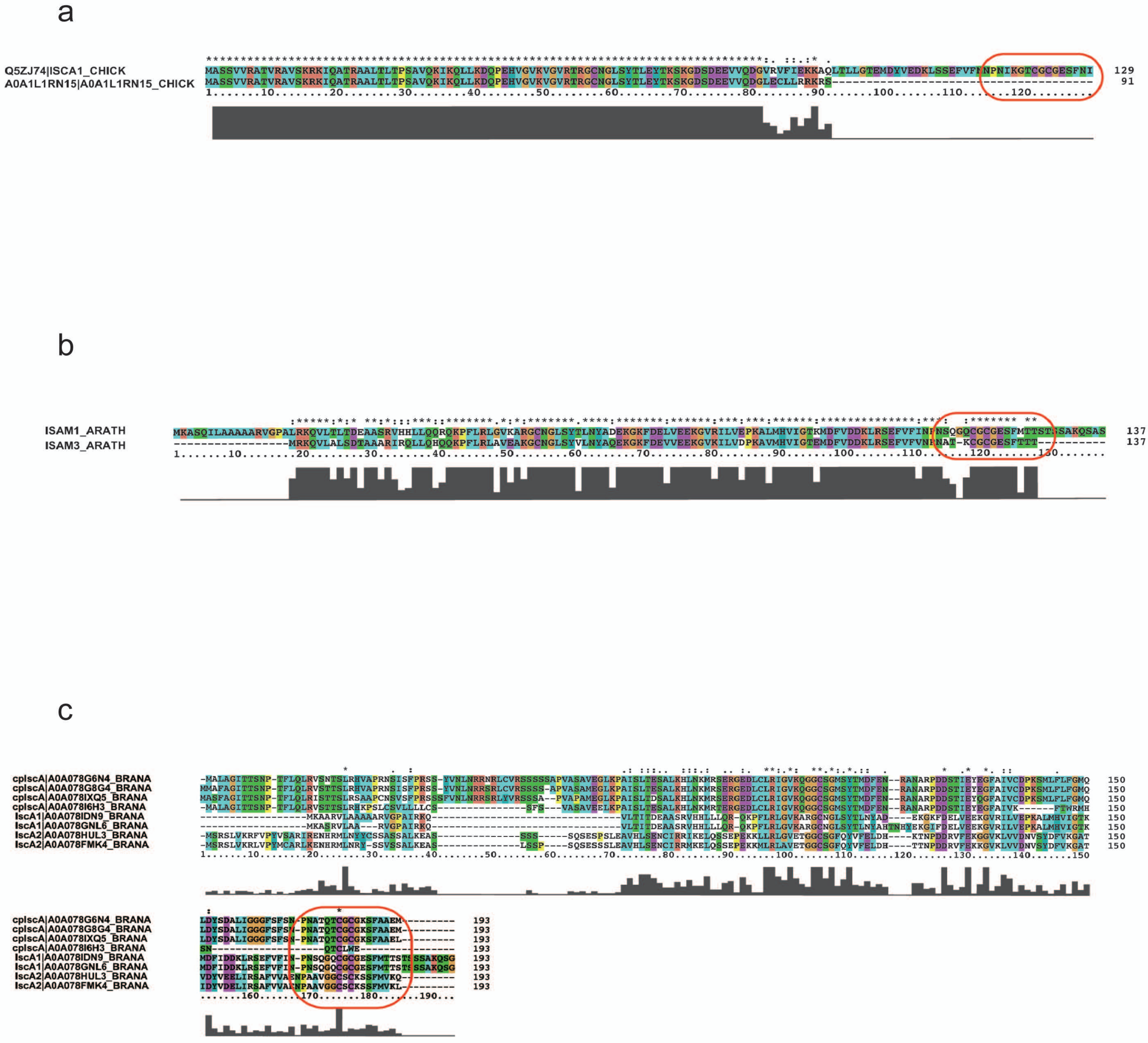

a
Q5ZJ74|ISCA1_CHICK
A0A1L1RN15|A0A1L1RN15_CHICK
b
ISAM1_ARATH
ISAM3_ARATH
c
cpIscA|A0A078G6N4_BRANA
cpIscA|A0A078G8G4_BRANA
cpIscA|A0A078IXQ5_BRANA
cpIscA|A0A078I6H3_BRANA
IscA1|A0A078IDN9_BRANA
IscA1|A0A078GNL6_BRANA
IscA2|A0A078HUL3_BRANA
IscA2|A0A078FMK4_BRANA

Figure S4

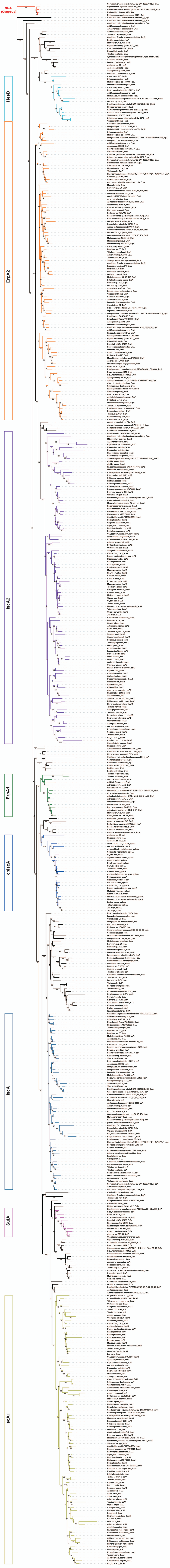
NfuA
(Outgroup)
HesB
ErpA2
IscA2
ErpA1
cpIscA
IscA
SufA
IscA1

Figure S5

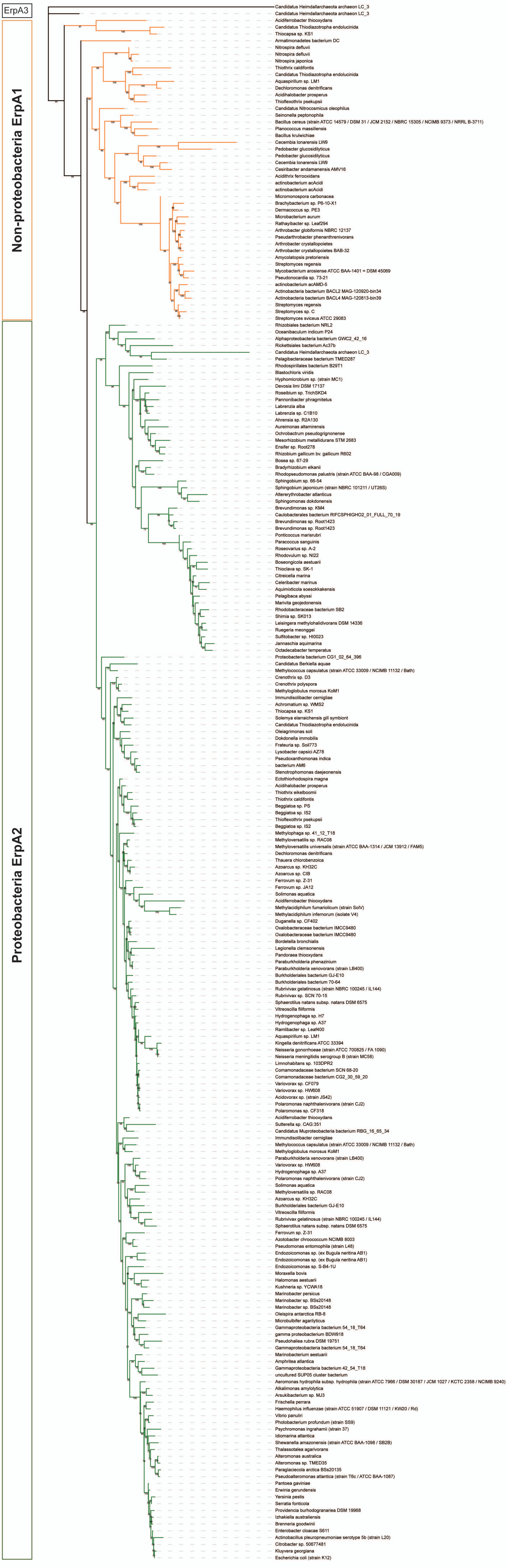

ErpA3
Non-proteobacteria ErpA1
Proteobacteria ErpA2
Candidatus Heimdallarchaeota archaeon LC_3
Candidatus Heimdallarchaeota archaeon LC_3
Acidiferrobacter thiooxydans
Candidatus Thiodiazotropha endolucinida
Thiocapsa sp. KS1
Armatimonadetes bacterium DC
Nitrospira defluvii
Nitrospira defluvii
Nitrospira japonica
Thiothrix caldifontis
Candidatus Thiodiazotropha endolucinida
Aquaspirillum sp. LM1
Dechloromonas denitrificans
Acidihalobacter prosperus
Thioflexothrix psekupsii
Candidatus Nitrocosmicus oleophilus
Seinonella peptonophila
Bacillus cereus (strain ATCC 14579 / DSM 31 / JCM 2152 / NBRC 15305 / NCIMB 9373 / NRRL B-3711)
Planococcus massiliensis
Bacillus krulwichiae
Cecembia lonarensis LW9
Pedobacter glucosidilyticus
Pedobacter glucosidilyticus
Cecembia lonarensis LW9
Cesiribacter andamanensis AMV16
Acidithrix ferrooxidans
actinobacterium acAcidi
actinobacterium acAcidi
Micromonospora carbonacea
Brachybacterium sp. P6-10-X1
Dermacoccus sp. PE3
Microbacterium aurum
Rathayibacter sp. Leaf294
Arthrobacter globiformis NBRC 12137
Pseudarthrobacter phenanthrenivorans
Arthrobacter crystallopoietes
Arthrobacter crystallopoietes BAB-32
Amycolatopsis pretoriensis
Streptomyces regensis
Mycobacterium arosiense ATCC BAA-1401 = DSM 45069
Pseudonocardia sp. 73-21
actinobacterium acAMD-5
Actinobacteria bacterium BACL2 MAG-120920-bin34
Actinobacteria bacterium BACL4 MAG-120813-bin39
Streptomyces regensis
Streptomyces sp. C
Streptomyces sviceus ATCC 29083
Rhizobiales bacterium NRL2
Oceanibaculum indicum P24
Alphaproteobacteria bacterium GWC2_42_16
Rickettsiales bacterium Ac37b
Candidatus Heimdallarchaeota archaeon LC_3
Pelagibacteraceae bacterium TMED287
Rhodospirillales bacterium B29T1
Blastochloris viridis
Hyphomicrobium sp. (strain MC1)
Devosia limi DSM 17137
Roseibium sp. TrichSKD4
Pannonibacter phragmitetus
Labrenzia alba
Labrenzia sp. C1B10
Ahrensia sp. R2A130
Aureimonas altamirensis
Ochrobactrum pseudogrignonense
Mesorhizobium metallidurans STM 2683
Ensifer sp. Root278
Rhizobium gallicum bv. gallicum R602
Bosea sp. 67-29
Bradyrhizobium elkanii
Rhodopseudomonas palustris (strain ATCC BAA-98 / CGA009)
Sphingobium sp. 66-54
Sphingobium japonicum (strain NBRC 101211 / UT26S)
Altererythrobacter atlanticus
Sphingomonas dokdonensis
Brevundimonas sp. KM4
Caulobacterales bacterium RIFCSPHIGHO2_01_FULL_70_19
Brevundimonas sp. Root1423
Brevundimonas sp. Root1423
Ponticoccus marisrubri
Paracoccus sanguinis
Roseovarius sp. A-2
Rhodovulum sp. NI22
Boseongicola aestuarii
Thioclava sp. SK-1
Citreicella marina
Celeribacter marinus
Aquimixticola soesokkakensis
Pelagibaca abyssi
Marivita geojedonensis
Rhodobacteraceae bacterium SB2
Shimia sp. SK013
Leisingera methylohalidivorans DSM 14336
Ruegeria meonggei
Sulfitobacter sp. HI0023
Jannaschia aquimarina
Octadecabacter temperatus
Proteobacteria bacterium CG1_02_64_396
Candidatus Berkiella aquae
Methylococcus capsulatus (strain ATCC 33009 / NCIMB 11132 / Bath)
Crenothrix sp. D3
Crenothrix polyspora
Methyloglobulus morosus KoM1
Immundisolibacter cernigliae
Achromatium sp. WMS2
Thiocapsa sp. KS1
Solemya elarraichensis gill symbiont
Candidatus Thiodiazotropha endolucinida
Oleiagrimonas soli
Dokdonella immobilis
Frateuria sp. Soil773
Lysobacter capsici AZ78
Pseudoxanthomonas indica
bacterium AM6
Stenotrophomonas daejeonensis
Ectothiorhodospira magna
Acidihalobacter prosperus
Thiothrix eikelboomii
Thiothrix caldifontis
Beggiatoa sp. PS
Beggiatoa sp. IS2
Thioflexothrix psekupsii
Beggiatoa sp. IS2
Methylophaga sp. 41_12_T18
Methyloversatilis sp. RAC08
Methyloversatilis universalis (strain ATCC BAA-1314 / JCM 13912 / FAM5)
Dechloromonas denitrificans
Thauera chlorobenzoica
Azoarcus sp. KH32C
Azoarcus sp. CIB
Ferrovum sp. Z-31
Ferrovum sp. JA12
Solimonas aquatica
Acidiferrobacter thiooxydans
Methylacidiphilum fumariolicum (strain SolV)
Methylacidiphilum infernorum (isolate V4)
Duganella sp. CF402
Oxalobacteraceae bacterium IMCC9480
Oxalobacteraceae bacterium IMCC9480
Bordetella bronchialis
Legionella clemsonensis
Pandoraea thiooxydans
Paraburkholderia phenazinium
Paraburkholderia xenovorans (strain LB400)
Burkholderiales bacterium GJ-E10
Burkholderiales bacterium 70-64
Rubrivivax gelatinosus (strain NBRC 100245 / IL144)
Rubrivivax sp. SCN 70-15
Sphaerotilus natans subsp. natans DSM 6575
Vitreoscilla filiformis
Hydrogenophaga sp. H7
Hydrogenophaga sp. A37
Ramlibacter sp. Leaf400
Aquaspirillum sp. LM1
Kingella denitrificans ATCC 33394
Neisseria gonorrhoeae (strain ATCC 700825 / FA 1090)
Neisseria meningitidis serogroup B (strain MC58)
Limnohabitans sp. 103DPR2
Comamonadaceae bacterium SCN 68-20
Comamonadaceae bacterium CG2_30_59_20
Variovorax sp. CF079
Variovorax sp. HW608
Acidovorax sp. (strain JS42)
Polaromonas naphthalenivorans (strain CJ2)
Polaromonas sp. CF318
Acidiferrobacter thiooxydans
Sutterella sp. CAG:351
Candidatus Muproteobacteria bacterium RBG_16_65_34
Immundisolibacter cernigliae
Methylococcus capsulatus (strain ATCC 33009 / NCIMB 11132 / Bath)
Methyloglobulus morosus KoM1
Paraburkholderia xenovorans (strain LB400)
Variovorax sp. HW608
Hydrogenophaga sp. A37
Polaromonas naphthalenivorans (strain CJ2)
Solimonas aquatica
Methyloversatilis sp. RAC08
Azoarcus sp. KH32C
Burkholderiales bacterium GJ-E10
Vitreoscilla filiformis
Rubrivivax gelatinosus (strain NBRC 100245 / IL144)
Sphaerotilus natans subsp. natans DSM 6575
Ferrovum sp. Z-31
Azotobacter chroococcum NCIMB 8003
Pseudomonas entomophila (strain L48)
Endozoicomonas sp. (ex Bugula neritina AB1)
Endozoicomonas sp. (ex Bugula neritina AB1)
Endozoicomonas sp. S-B4-1U
Moraxella bovis
Halomonas aestuarii
Kushneria sp. YCWA18
Marinobacter persicus
Marinobacter sp. BSs20148
Marinobacter sp. BSs20148
Oleispira antarctica RB-8
Microbulbifer agarilyticus
Gammaproteobacteria bacterium 54_18_T64
gamma proteobacterium BDW918
Pseudohaliea rubra DSM 19751
Gammaproteobacteria bacterium 54_18_T64
Marinobacterium aestuarii
Amphritea atlantica
Gammaproteobacteria bacterium 42_54_T18
uncultured SUP05 cluster bacterium
Aeromonas hydrophila subsp. hydrophila (strain ATCC 7966 / DSM 30187 / JCM 1027 / KCTC 2358 / NCIMB 9240)
Alkalimonas amylolytica
Arsukibacterium sp. MJ3
Frischella perrara
Haemophilus influenzae (strain ATCC 51907 / DSM 11121 / KW20 / Rd)
Vibrio panuliri
Photobacterium profundum (strain SS9)
Psychromonas ingrahamii (strain 37)
Idiomarina atlantica
Shewanella amazonensis (strain ATCC BAA-1098 / SB2B)
Thalassotalea agarivorans
Alteromonas australica
Alteromonas sp. TMED35
Paraglaciecola arctica BSs20135
Pseudoalteromonas atlantica (strain T6c / ATCC BAA-1087)
Pantoea gaviniae
Erwinia gerundensis
Yersinia pestis
Serratia fonticola
Providencia burhodogranariea DSM 19968
Izhakiella australiensis
Brenneria goodwinii
Enterobacter cloacae S611
Actinobacillus pleuropneumoniae serotype 5b (strain L20)
Citrobacter sp. 50677481
Kluyvera georgiana
Escherichia coli (strain K12)
